# Morality in the anthropocene: The perversion of compassion and punishment in the online world

**DOI:** 10.1093/pnasnexus/pgae193

**Published:** 2024-06-11

**Authors:** Claire E Robertson, Azim Shariff, Jay J Van Bavel

**Affiliations:** Department of Psychology, New York University, New York, NY 10003, USA; Department of Psychology, University of British Columbia, Vancouver, BC V6T 1Z4, Canada; Department of Psychology, New York University, New York, NY 10003, USA; Department of Neural Science, New York University, New York, NY 10003, USA; Department of Strategy & Management, Norwegian School of Economics, Bergen 5045, Norway

**Keywords:** morality, online mismatches, compassion, punishment and shaming

## Abstract

Although much of human morality evolved in an environment of small group living, almost 6 billion people use the internet in the modern era. We argue that the technological transformation has created an entirely new ecosystem that is often mismatched with our evolved adaptations for social living. We discuss how evolved responses to moral transgressions, such as compassion for victims of transgressions and punishment of transgressors, are disrupted by two main features of the online context. First, the *scale* of the internet exposes us to an unnaturally large quantity of extreme moral content, causing compassion fatigue and increasing public shaming. Second, the physical and psychological *distance* between moral actors online can lead to ineffective collective action and virtue signaling. We discuss practical implications of these mismatches and suggest directions for future research on morality in the internet era.

## Morality in the anthropocene: the perversion of compassion and punishment in the online world

Just as the atomic bomb changed how nations conduct warfare and the birth control pill changed how people have sex, the internet has changed moral psychology. The human tendency to care about moral issues like fairness, reciprocity, and empathy were evolutionarily adaptive for improved functioning in small, close-knit societies where people directly relied on their close social ties to survive (1–3). Today, the environment people inhabit is undergoing a shift that is arguably larger than that of the agricultural revolution 12,000 years ago. Estimates suggest that over 5 billion people (over 60% of the entire world) use the internet regularly (4). This number is much higher in developed countries, where rates of regular use are as high as 99%, making the experience of the internet nearly universal in some cultures (5). In this article, we explain how the internet disrupts humanity's basic moral instincts. Our review explains how people's evolved moral psychology makes it easy to exploit them with algorithms, endless newsfeeds, and outrageous content.

**Fig. 1.**
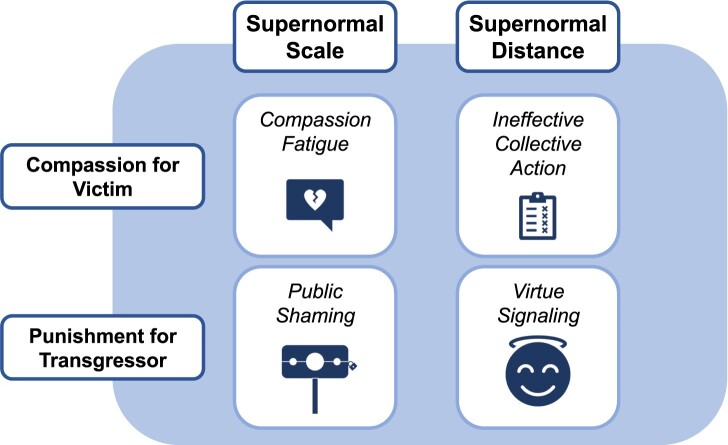
Visual representation of the framework for how the scale and distance afforded by the internet distorts our evolved reactions for compassion for victims and punishment of transgressors in moral interactions. *Top left*: When the supernormal scale of the internet interacts with people's instinct to feel compassion for victims of moral transgressions, it can result in compassion fatigue. *Top right*: when the supernormal distance of the internet interacts with people's instinct to feel compassion for victims of moral transgressions, it can result in ineffective collective action. *Bottom left*: When the supernormal scale of the internet interacts with people's instinct to punish moral transgressors, it can result in public shaming. *Bottom right:* When the supernormal distance of the internet interacts with people's instinct to punish moral transgressors, it can result in virtue signaling.

The shift to the online environment fundamentally changed the social world, and we argue that evolved behaviors that were advantageous in small groups are often poorly suited to navigate the online environment. Evolved responses to moral conflict between group members, like compassion for the victim and punishment for the transgressor, have different outcomes online than they do in small groups. Here, we discuss how the socially functional outcomes of compassion and punishment are disrupted online by two main features of the online context. First, the *scale* of the internet exposes us to an unnaturally large quantity of extreme moral content. Online, people are exposed to moral content in greater quantities and of greater intensity than they are offline, causing dysfunctional outcomes like compassion fatigue and increasing public shaming. Second, the physical and psychological *distance* between moral actors online makes people's reactions to moral transgressions evolutionarily mismatched. The increased distance between punishers and transgressors online shifts the dynamics of punishment from their evolutionary optima, leading to ineffective collective action and virtue signaling. These mismatches play a role in increasing negativity, outrage, and intergroup conflict (Fig. 1).

## Evolutionary underpinnings of moral cognition

Humans are a highly social species (6), and much of the evolved, innate behaviors that humans possess are related to navigating social situations (3, 7–10). People are far more likely to both survive and thrive when they have strong social connections (11). Thus, morality is hypothesized to have evolved due to early humans' need to effectively cooperate with fellow group members and navigate social relationships (3, 12). Violations of cooperative relationships—be it through causing harm, failing to reciprocate, or betraying obligations to a family or group—are seen as morally transgressive (3). The quick recognition of and reaction to moral stimuli is functional, especially in the context of evolutionary adaptation of humans' ancestors (13). In small group contexts, communities of individuals who are predisposed to detect and react negatively to violations of care and cooperation norms are likely to build stronger and more successful groups over time (14–16). A tendency to avoid causing suffering to others and to punish those who cause others suffering bestowed fitness benefits by increasing reciprocity, reducing in-group violence, and signaling positive parental traits. Thus, preferentially attending to moral stimuli elicited helpful and protective behavior, and continues to this day (9, 17–20).

As society became more complex, so too did people's conceptualization of and reasoning about morality. Today, moral reasoning depends on culturally specific norms (21, 22), and occurs via complex cognitive systems by which people blend emotionality and rationality, take context and intentionality into account, and make utilitarian judgments when necessary (23–25). Moreover, it is regulated and guided by institutions and elected third parties (26). Nonetheless, vestiges of people's evolved instincts remain and continue to influence moral cognition and decision making (27–29). For instance, attention towards morally relevant stimuli is hard to suppress—as people recognize morally relevant stimuli more quickly and more consistently than other types of stimuli (30, 31). Other research suggests that moral and emotional language capture early visual attention better than neutral content (32). Thus, people seem to have an attentional preference for content that signals moral relevance.

## The internet and supernormal moral stimuli

The modern era of the anthropocene—the epoch of time in which humans have been the dominant force in the global environment (33, 34)—has been likewise marked by a substantial change in the size and complexity of human social networks (35). For almost 99% of our species' history, humans lived in small, nomadic tribes—a state that characterized what is commonly referred to as our Environment of Evolutionary Adaptedness (36). With the Pleistocene–Holocene transition roughly 12,000, humans began to shift away from this state—moving to settled agricultural communities, to market-based economies, and eventually into a communication age driven by recent technologies such as newspapers, telephones, and televised mass media. But the shift to the internet in the last 30 years has fundamentally changed the scale of social interactions and information (37). Unlike the post and telephones which connect people one-to-one, or newspapers and mass media which connect people one-to-many, the internet is the first technology that allows for connections of the many to the many with no concern for time or distance. It has fundamentally changed the way people all over the world communicate with one another. Moreover, it has introduced an entirely new environment—one not just dominated, but wholly created, by human beings.

The internet now connects over 5.3 billion people around the world (38). People spend an average of almost 7 hours per day online, almost as much as the time spent sleeping (39). In those 7 hours, people consume a massive amount of content: data from Facebook suggest that people scroll through roughly 300 feet of content a day, or almost the height of the Statue of Liberty (40). This amount of content is equivalent to reading every page of *The New York Times* more than three times over. It is also orders of magnitude larger than the single newssheets that represent the first iterations of newspapers in the United States in the early 18th century (41). This content comes from many people across distributed social networks that are much larger than previous estimates of historical social network size (8).

**Fig. 2.**
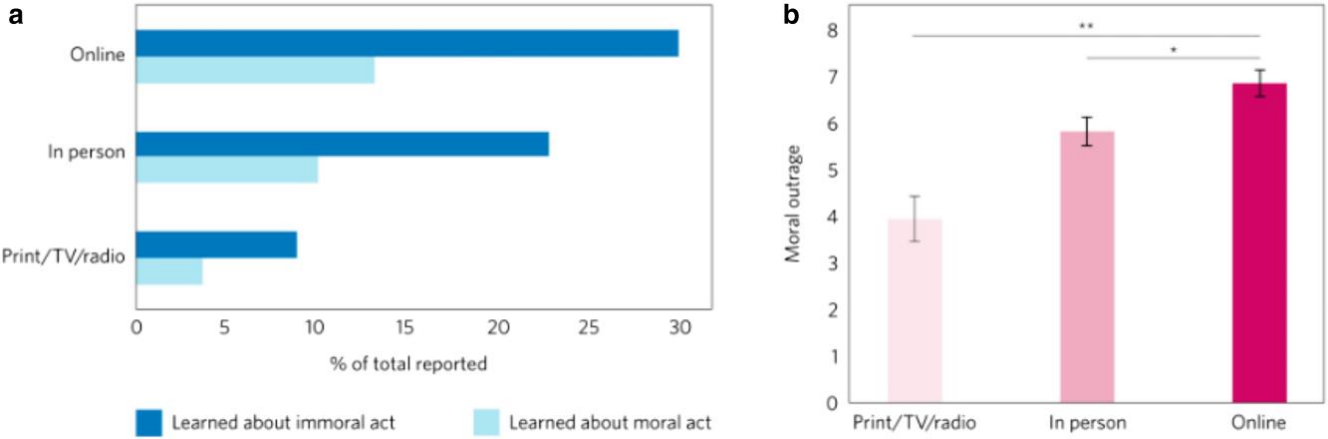
In a large sample of North American adults, a) People were more likely to learn about immoral acts online than in person or via traditional forms of media (print, television, and radio). The figure displays the percentage of total reported moral/immoral acts that were learned about in each setting. b) Immoral acts encountered online evoked more outrage than immoral acts encountered in person or via traditional forms of media. Error bars represent SEM (Figure adapted from Ref. (43)).

Much of the activity that people engage in online relates to social goals (42). As people are exposed to more social content in general, the rate of moral content people are exposed to is also increasing. For instance, people are significantly more likely to learn secondhand about an immoral event in an online context than from print, radio, and TV combined (43) (Fig. 2). This is a striking difference from the infrequency of morality in everyday conversations (44) and underscores the centrality of morality online. We describe two factors that exploit people's attention towards morality in the online environment: overabundance and extremity.

### Overabundance

The overabundance of moral content online is likely related to people's attentional preference towards morally relevant stimuli (13). In the attention economy, moral content often generates the greatest engagement (45). For example, tweets that contain moral–emotional language have a greater likelihood of being shared than neutral tweets (46)—this is true for tweets by both lay people and political elites (47). Similarly, news stories that are framed morally receive more shares than neutral news stories online (48). Moreover, the same moral and emotional words that capture attention in controlled lab settings are also more likely to be shared (i.e. retweeted) within real social media contexts (49). Consequently, these results suggest that the attention-grabbing nature of moral and emotional words contributes to the accelerated spread of moral content on social media platforms.

Overabundance of stimuli across many domains can have cognitive consequences due to the way humans detect and summarize information about others. When a target stimulus is presented rarely, people tend to miss the actual appearance of a stimuli. However, when the target is presented with overabundance, people tend to report the target even when it is not there (50). In an online environment saturated with moral transgressions, this could lead people to perceive transgressions even when there are none present. Moreover, moral content is prioritized in visual attention, and this predicts online engagement (32). As such, overexposure to moral content might shape behavior in numerous ways.

Regarding information summation, people weigh negative information more heavily than positive information about a person (51). As people summarize information about another person's moral character, negative moral information has a stronger effect on perception of character than positive information (52). Additionally, when people are given many unique exemplars to remember, they engage in a process called ensemble coding, by which they take the average of a series of stimuli based on certain traits (53, 54). However, ensemble coding can be biased by the most extreme or unexpected exemplars in a group (55, 56)—a particular problem online and on social media platforms, where people with the most extreme views generate the most content (57). Indeed, 97% of political posts from Twitter/X come from just 10% of users, meaning that roughly 90% of the population's political opinions are being represented by less than 3% of posts online (58). This reveals how information summation may be misled by the overabundance of information online, leading to biases towards negative moral evaluations or the generation of extreme false norms.

### Extremity

The moral content people are exposed to online is often more extreme than typical moral content. The immoral acts that people learn about online tend to elicit stronger feelings of outrage compared to the events that are witnessed in person (43). This suggests that the immoral acts learned online tend to be more extreme than immoral acts encountered in person. One way to think about the effects of heightened extremity of moralized content online is through the lens of *supernormal stimuli.* Supernormal stimuli mimic the stimuli in the environment that organisms are predisposed to preferentially attend to, but are more extreme than they would ever be in the natural environment (42, 59–62). For example, modern fast food is considered to be a supernormal stimulus (63). People evolved to seek out fatty and calorically dense foods, as those types of foods were more likely to help sustain people through periods of relative scarcity that were prevalent in humans' evolutionary history. However, in the modern era of the anthropocene, most people live in relative abundance, and people's tendency towards fatty foods now contributes to people overeating unhealthy foods, leading to heart disease, diabetes, and other health complications. Extreme moralized content online may function in a similar way, capturing our attention and triggering unhealthy behavior against our better judgment.

Recently, Bor and Peterson (64) argued that the mismatch hypothesis does not explain online hostility. They note that people are consistent in their levels of hostility both online and offline, suggesting that online contexts do not change people's hostility, but simply enhance the visibility of people who are already hostile. We argue, however, that the increase in visibility makes the online environment a more routinely hostile and extreme place, potentially creating a mismatch with people's experiences in the real world where such hostility is less visible. Most social media content is produced by a small subset of users who tend to be the most ideologically extreme and the most active online (58,65). Indeed, those who have the strongest negative feelings about a group or topic are also the most likely to share negative content online (66). This may lead the online environment to be saturated by the extreme content posted by those who, in turn, hold the most extreme opinions.

This feature of the online world can artificially inflate people's perceptions of animosity and outrage—creating false norms (49). This may be further distorted because people engage in both homophily where they choose to connect with individuals who are ideologically similar to them (46, 67)—and acrophily where they choose those who share their ideology but are slightly more extreme than them (68). Thus, people's social networks tend to be flooded with opinions that are, on average, more extreme than their own opinions or the opinions they experience in the real world. This is further exacerbated by both algorithms and social reinforcement learning (49, 69). This is a cyclical process: algorithms are built to maximize engagement online, and the people who engage the most are also those with the most extreme opinions (70). Thus, algorithms “learn” that the most extreme content is the most successful at garnering online engagement, and prioritize that type of content—even if people do not like it (see Ref. (71)). We argue that two of the outcomes of this cyclical increase in extreme content are the disruption of compassion and third-party punishment online.

In summary, we theorize that the overabundance and extremity of online content lead people's evolutionary moral dispositions to be perpetually triggered. This, in turn, increases the production and spread of moral content online—further feeding a morally saturated environment. In the next two sections, we examine two areas of moral cognition—compassion and third-party punishment—to illuminate how the internet and social media exploits basic moral cognition, eliciting behavior that is maladaptive for both individuals and society.

## Compassion and empathy

### Offline and online

It is natural to feel compassion and empathy for victims. In reaction to witnessing a moral transgression, people feel compassion, empathy, and a desire for restitution for the victim (9, 72, 73). Empathy spurs action—groups whose members can empathize and have compassion for others are more likely to take care of each other and of vulnerable offspring, increasing the odds of survival and gene propagation (9, 74). In modern times, empathy is associated with higher donations to charity and those in need (75, 76). However, the compassion that humans evolved to feel for victims is altered due to the distance between social ties online.

Despite these benefits, people are selective in whom they empathize with (77). People are more likely to empathize with in-group members compared to out-group members (78, 79) and less likely to feel empathy for more distant social connections (80). This is because empathizing can be emotionally taxing, and people will avoid it when possible (81). Moreover, empathy is a costly cognitive resource, and people want to reserve it for those who may be able to help them at a later time, such as in-group members (20). Thus, the limits of empathy are regularly tested in online contexts, where people are exposed to supernormal levels of moral content from distant and loose social connections.

When people are overloaded with requests for empathy, people find assigning blame easier than having empathy (82). Online, this may lead to people reacting to transgressions to focus on assigning blame rather than empathizing with a victim. This is especially problematic, since one of the most effective ways to reduce hateful speech online is to express empathy (83). When comparing online empathy and offline empathy directly, offline empathy is significantly stronger than online empathy (84), suggesting that people may morally disengage online, relieving themselves of the responsibility to act (85). Taken together, this evidence suggests that people are less likely to feel compassion and act in restorative ways, and more likely to assign blame to victims when confronted with the supernormal quantities of suffering that are typical of online engagement.

### Supernormal scale and compassion fatigue

The tendency to feel compassion towards the victims of a moral transgression does not scale well online due to the high exposure to victims. People respond with more empathy to a single victim than to a group of victims (86, 87). They become numb to excess suffering and do not linearly scale their empathy with the number of victims. For example, people are willing to donate roughly the same amount of money to help from 2,000 to 200,000 victims (88). This may be in part because people are averse to taking on too much responsibility for large numbers of moral victims (89). Indeed, when there are many victims rather than just a few, people are motivated to disengage from a conflict and not act (90). As the number of victims in a scenario increases, the likelihood that people will take prosocial action like donating money actually goes down (90). This may be related to processes by which, when an experience is common, people value it less over time than when it is rare (91). For example, overexposure to moral transgressions can have a numbing effect on observers. When people are repeatedly exposed to the same information about a moral transgression, they later report that that transgression seems less unethical than a novel transgression (92). This may lead them to feeling that the transgression was “not that bad” and therefore reduce their compassion for a victim.

Even when people do choose to behave prosocially online, their actions often make little to no real impact. This may be because of moral licensing, or the belief that a prior good deed “licenses” a person to engage in morally questionable behavior later (93). For example, engaging in a noncostly form of compassion, such as “liking” or “sharing” a post, may lead people to believe that they have absolved themselves of their moral responsibility to engage in further prosocial action (94, 95). Indeed, the common tagline of “thoughts and prayers,” often posted online after disasters in the United States, may undercut monetary donations to those in need (95). There are exceptions to this—the Ice Bucket Challenge, for example, raised millions of dollars for ALS research, and relied on people's desires to share prosocial information online (96). In most other cases, however, low-cost forms of prosocial behavior can, ironically, hinder the material impacts of positive social movements. Thus, an evolved tendency for compassion and empathy can lead to a decrease in overall prosocial behavior when in an online context.

### Supernormal distance and ineffective collective action

In rare cases, mass sharing can be helpful. Internet use has been credited with spawning protests and demonstrations of collective action around the globe such as the Arab Spring and Black Lives Matter (97, 98). The internet has indisputably increased broad awareness of a wide variety of social issues. The virality and traction these issues received, especially as they gave suppressed voices who may have been typically ignored by mainstream media an outlet to collectively organize and share experiences (99), created broad awareness that would have been impossible without the internet. Unfortunately, while awareness of social issues is often a net positive, it does not directly translate to increased action towards fixing an issue. Indeed, there is increasing debate about how (in)effective online activism really is (100, 101). For instance, even though social media-driven nonviolent protests are larger now compared to most historical protest movements, they have resulted in far less policy change (102). This may be in part because of increased psychological distance between individuals who participate in online activism (101, 102). This has led to broad but shallow interest in these causes which may actually harm the causes in the long run (103) and foster cynicism.

It is theorized that this drop in efficacy is because activism used to require deep roots and structures to get off the ground, stronger dedication to a cause, and months of planning to execute (101). This led to vibrant social networks and clear organizational goals. Now protests can be organized within a matter of days due to social media, potentially leading more people to show up to a protest (98). However, many of those who attend protests are less dedicated to the cause or the group than they would have been the case historically. Moreover, their engagement might be motivated by superficial self-interest (e.g. creating online content to signal an affiliation to gain social status). Thus, while online activism may increase awareness of inequities or social problems, it can actually hinder the effectiveness of collective action by prioritizing shallow, low-cost forms of collective action that are not effective at convincing or pressuring those in power to make lasting policy change (101, 102, 104).

## Third-party punishment

### Offline and online

In addition to compassion for the victim, witnessing a moral transgression also spurs punishment towards the transgressor. Like compassion fatigue, the desire to punish a wrongdoer often occurs when one is a third party to a moral transgression. In fact, people are most punitive when they are mere bystanders to a moral transgression (105). The drive to engage in costly third-party punishment—or the act of punishing wrongdoers even when that punishment comes at a personal cost—appears to be a culturally universal, likely evolved, tendency (7, 18, 106). Research using economic games has found robust evidence of third-party punishment (107). The motivation to punish transgressors emerges early in development, as young children engage in costly punishment towards in-group and out-group moral transgressors (108) giving up a treasured resource (being able to use a slide) in order to punish other children who behaved immorally (109). Evolutionarily, costly third-party punishment may have developed in small groups to deter cheating and freeriding behavior, thus strengthening the group over time (7, 14, 15).

On the surface, third-party punishment is an evolutionary puzzle: why would it be beneficial to sacrifice one's own resources to punish a bad actor, especially when one is not personally harmed? One clue is that third-party punishment is only effective when people are required to cooperate with the same group repeatedly (14). Punishment is not as effective when the makeup of groups changes, or in one-shot dilemmas. Furthermore, punishment increases cooperation and group resource contributions most when it is done in public, or in full view of the rest of the group (110). This suggests that social shame acts as a deterrent for bad behavior among in-group members, in addition to any material loss incurred as punishment. Additionally, publicly rebuffing someone helps the punisher by deterring future cheaters (110). Thus, punishment as a response to witnessing moral transgressions highlights deep-rooted motivations to punish wrongdoers and uphold fairness in social interactions.

In addition to punishing cheaters to deter future immoral behavior, engaging in third-party punishment may confer reputational benefits to the punisher (111, 112). Indeed, people are more likely to engage in third-party punishment when they have an audience (19). Part of the reason that third-party punishment is effective at maintaining group cohesion is that it signals commitment to one's group and re-establishes that commitment as a group norm. To be effective, it requires a real sacrifice, either in resources or in personal risk, to the group for the sake of justice (15, 18, 110, 113–115). Thus, engaging in third-party punishment makes someone a more attractive mate or cooperation partner, as it signals trustworthiness and willingness to sacrifice for others (16). Indeed, engaging in costly third-party punishment demonstrates moral fiber to one's group members, and can lead to admiration and increased status in the eyes of observers (110–112). Computer models of evolving group dynamics found that group members who remained in “good standing” reputationally (i.e. helped others when they could) propagated their genes more easily over time (10).

### Supernormal scale and public shaming

When this tendency to punish moral wrongdoers is engaged in the online context, it has unexpected consequences. As the number of possible third-party punishers increases, the average third-party punishment intensity decreases only mildly, leading to a substantial increase in total punishment as group size increases (116). When people learn of a moral transgression online, they have an urge to punish the transgressor, just as was the case when punishment occurred in small groups. However, online interactions do not take place within a small group. On the contrary, many instances of online shaming or punishment involve one transgressor being punished by thousands of people, most of whom have no offline relationship with the transgressor (117). People in online communities are not required to work or live together at any point, because they are geographically spread apart and do not visibly rely on one another to fulfill day to day tasks. Online, groups function more to signal belonging to a specific social identity such as political party. The superficiality of these connections to relatively unknown strangers can lead people to have black-and-white judgments of morality with little nuance (118). This can lead to a massive campaign of retribution against a complete stranger.

Due to the massive scale of online social networks, the population from which third-party punishment can spring is immense. Instead of a small tribe of people who have a vested interest in fostering group cooperation, millions of people from anywhere in the world can gather to publicly punish one person with no personal investment or genuine desire for restitution. They might seek to gain social status without any genuine attempt to improve collective outcomes. Throughout evolutionary history, third-party punishment was usually administered by people who had a stake in the outcome, and also typically by in-group members. Indeed, the likelihood that a third-party observer would eventually have to interact with either the transgressor or the victim of a moral transgression was extremely high (119, 120). However, in online contexts there are millions of third-party observers, and very few, if any, will ever meet a particular transgressor in real life. This can undercut the traditional social function of cooperation and incentivize activities like public shaming that are disproportionate to the original transgression. Punishment in this context focuses on exacting retribution instead of rehabilitation or education.

### Supernormal distance and virtue signaling

Physical distance between the punisher and the punished means that online shaming and punishment is rarely costly to the punisher (43). Thus, punishing people online is not an effective signal of group commitment or trustworthiness. As such, third-party punishers may engage immoral grandstanding or selfish virtue signaling (121, 122). In the online environment, virtue signaling refers to a type of false signaling where people publicly claim to be morally virtuous to enhance their own moral reputation, without exemplifying that virtue in a meaningful way (123). Online, there is near endless evidence of out-group members behaving badly, allowing in-group members many opportunities to signal their status as “good group member” and respond virtuously, inadvertently escalating the conflict (124). This can undercut the core function of costly punishment by making it cheap enough for noninvested strangers to participate.

Importantly, people can signal their true moral beliefs on social media. However, virtue signaling is often seen as hypocritical in online contexts because the signaler received social rewards (i.e. likes/shares) for saying the “right thing” without requiring the signaler to actually “do the right thing” (125). Thus, when moral outrage and shaming goes viral, and thousands of people costlessly reprimand a single transgressor, outside observers are less likely to see that outrage as genuine (126). Instead, people perceive punishers as bullies when they are part of a large group of online punishers and begin feeling empathy for the original transgressor. Hence, people's evolutionary motives to punish moral transgressors may have an inverse effect from their evolved function: rather than signaling that one is just and righteous, others may perceive their virtue signaling as a sign of immorality (126) or disingenuousness (127). Thus, online public shaming can have the opposite effect from its evolutionary roots, reducing trust in punishers and increasing sympathy for transgressors. It may also foster genuine cynicism about the actors or about online moral discourse.

Regarding the shaming and punishing of a moral transgressor, the evolutionary mismatch of punishment tendencies in the new online context changes the outcome of punishment. In addition to increasing the status of a punisher, third-party punishment also served evolutionarily to deter cheaters from transgressing again (128, 129). However, the deterring effects of punishment and shaming worked best when engaged in small groups who would have repeated interactions over time (14, 130). The online context is different in both of these regards. Due to the extremely high rate of relational mobility (i.e. the frequency and flexibility by which people are able to encounter new social partners, and form and end social relationships) that people experience online, they are easily able to move out of one group and into a new group with all new social participants (131–133). As a result, punishing transgressors may not successfully deter repeated wrongdoings when executed online. Therefore, people feel more at liberty to say or do things online that they would not say in real life (134, 135).

Furthermore, public shaming transgressors may actually increase their negative feelings and resentment towards punishers, rather than guilt over their transgressive actions (136, 137). This may lead transgressors to focus on the proportionality of their transgression compared to the reaction of the public, rather than on changing their behavior (117). This can lead to the continuation or escalation of conflict. Transgressors might even develop communities around these grievances and seek revenge. Thus, the shifting dynamics of the online realm, characterized by high relational mobility and the perception of punishers as bullies, reduce the effectiveness of punishment as a deterrent against repeated wrongdoings.

## Future directions

We have presented several clear examples where we think mismatches lead to surprising patterns of behavior. Research is now needed to test whether the assumptions made by the mismatch hypothesis are supported by empirical evidence. For example, if people engage in public shaming in order to reap reputational benefits from engaging in costly punishment (16), do people also go out of their way to signal that their online punishment was somehow costly to them despite the distance between themselves and the punished? Furthermore, if people engage in ineffective prosociality online to morally license themselves to disengage from mass suffering (89, 95), do people feel less empathy for victims of moral transgressions after they have been given a costless opportunity to express compassion on social media? Relatedly, given the ephemeral nature of online activism (101, 103), did movements that called for a long-term offline commitment to a cause, such as a boycott of a product or store, result in greater behavioral and psychological commitment to the cause compared to causes focusing on shorter offline commitments, such as protests? With these insights, researchers can begin to develop interventions to reduce negative individual and societal outcomes related to compassion and punishment mismatches online.

Part of the problem with reducing the mismatch between evolved moral behavior and the online environment is that the attention economy upon which the internet is built is currently structured to incentivise supernormal stimuli (45, 138, 139). The online environment is owned and regulated by a number of technology companies whose primary profits come from advertising (140). Advertising requires that people are engaging on a specific platform, and tech companies must compete for user attention (140). Considering that moral content often receives preferential attention (32), it is logical for companies to capitalize and promote moral content. There is little financial incentive for companies who profit from attention capture to reduce the use of supernormal stimuli on their platforms. For instance, interventions that reduce one's exposure to toxicity online also reduce engagement on social media sites (141). This undercuts the profitability of these platforms. Thus, it is unlikely companies will be motivated to change the online context in ways that ameliorate these evolutionary mismatches (without government regulation). On the contrary, we think it is more likely that companies will continue to exploit these tendencies as long as it remains profitable.

As such, future research should test platform design features that sustain attention or engagement without inducing negative externalities on individuals and society. There is evidence that people have a desire to make the internet a more positive place but lack the means to do so on their own. When asked directly, most online media users say that they want lower levels of outrage and negativity in their online feeds (71). Thus, allowing people to more easily regulate the types of content they do and do not want to see may reduce people's baseline exposure to morally outrageous content (139). Other design changes, such as allowing people to publicly signal their “trust” of a particular news story as an alternative to “liking” or “sharing” news, may help reduce the spread of misinformation by downregulating attention-grabbing and morally stimulating headlines (138). More research is needed on these prosocial design features.

Future research should focus on the longitudinal effects of overexposure to moral information online, especially looking at individual differences. Prior work examining individual-level outcomes in overexposure to the internet and social media have found that, while social media use can be positive for some people, it can have negative effects for vulnerable or at-risk populations (142). Moral discourse online is linked to subsequent violence in the real world (143). Furthermore, the internet has been a boon for hate groups–allowing them to flourish and organize extremists (144). Critically, even though certain conspiracy theories may originate online, they often bleed into the offline world, causing extremism, harm, and even death (145, 146).

While these studies have been correlational, large-scale experiments have found that limiting social media causes improvements in subjective well-being (e.g. (147, 148)). Thus, researchers should examine whether full social media cessation is required for well-being improvements, or if removal or reduction of specific content such as extreme content or users, could allow people to continue using social media while still improving their social interactions and well-being. Much is still unknown about the long-term effects of overexposure to negative information online.

It is difficult for researchers to effectively study the online environment because tech companies are reluctant to share how their algorithms function (149). This is true even though there is widespread agreement among lay people that greater transparency about social media algorithms (71). This critical lack of understanding has hindered scientists' abilities to critically examine the effects of social media on emotion and behavior (140, 150). Therefore, it is imperative that researchers have greater access to these algorithms to develop a better understanding of how they function. Ideally, stakeholders (e.g. users and members of the public) should also have input into algorithms that impact their lives.

We acknowledge that both the effect of social media and evolutionary theory are hard to test experimentally. One cannot assign people to have zero exposure to social media, or to acquire a specific evolutionary adaptation. Instead, much research on the effects of social media are correlational, or rely on natural experiments from archival data (for an example, see (151)). In order to drill down on the causal and evolutionary mechanisms that contribute to the mismatch of moral instincts online, researchers should consider more ambitious methods. Causal social media studies, such as cessation studies, have been effective in the past at investigating social media's effect on polarization in the United States (147) and Bosnia and Herzegovina (148). However, in order to argue that a trait is evolved and not learned, there must be evidence of that trait across cultures. Global collaborative efforts to replicate these studies and examine a wider range of outcome measures, including moral outrage and extremism, are already underway.^a^

Although we have focused on the areas where the distance between the traditional offline environment and the new online one has undermined the effectiveness of compassion and punishment, the internet is obviously not all bad. For example, the scale of the internet has raised the ceiling and lowered barriers for nearly every type of human knowledge, from simple online tutorials for learning new skills (152) to crowdsourcing solutions to our most difficult and pressing scientific conundrums (153). Furthermore, although people are more distant from those in their social groups, the internet has also brought together new social groups that could never have existed before, such as support groups for people with rare diseases who would have been unlikely to find each other in the real world due to physical distance (154, 155). While these benefits may be clear and demonstrable, however, the internet has also led to unexpected but consistent consequences that must be investigated as well. While small support groups may be positive forces in the lives of their users, why do large-scale social movements that originate online often stagnate (101, 102)? Why, when high quality knowledge is now universally available, does fake news proliferate online (156, 157)? Why are social media users willing to pay to have others—including themselves—deactivate these popular social media platforms (i.e. TikTok and Instagram; (158))? Understanding how the structure of the online environment can lead to such negative outcomes is the crucial first step in developing interventions and solutions to mitigate those negative outcomes.

## Conclusion

The changes that the internet has caused to our social environment have been larger and faster than any cultural or technological shift in our history. Humans are left using brains tuned for an offline world to navigate a novel environment of extreme stimuli and connectedness. However, humans have also evolved to be keen social learners and remarkably adaptable (159). Understanding how the internet can distort our moral instincts will help us navigate and shape our new environment and help prevent maladaptive outcomes for individuals and society.

## Notes

a
https://globalsocialmediastudy.com/

## Preprints

This manuscript was posted as a preprint: doi:10.31234/osf.io/ns34y.
